# Self‐Assembly, Rearrangement, and Disassembly of {Cr_6_} Horseshoe Oligomers

**DOI:** 10.1002/anie.202510610

**Published:** 2025-10-05

**Authors:** Niklas Geue, Dhaneesh Kumar, Jimin Ham, Shengpeng Huang, Grigore A. Timco, Neil A. Burton, Richard E. P. Winpenny, Kelvin Anggara, Perdita E. Barran

**Affiliations:** ^1^ Michael Barber Centre for Collaborative Mass Spectrometry Manchester Institute of Biotechnology Department of Chemistry The University of Manchester 131 Princess Street Manchester M1 7DN UK; ^2^ Nanoscale Science Department Max Planck Institute for Solid State Research Heisenbergstr. 1 70569 Stuttgart Germany; ^3^ Department of Chemistry The University of Manchester Oxford Road Manchester M13 9PL UK

**Keywords:** Collision‐induced dissociation, Ion mobility mass spectrometry, Ion soft‐landing, Mass spectrometry, Polymetallic compounds, Scanning probe microscopy

## Abstract

Molecular assemblies are commonly found in biological systems, and designing synthetic mimics of these is a challenge for modern chemistry. Here, we apply ion mobility mass spectrometry (IM‐MS), density functional theory (DFT), and mass‐selective electrospray ion beam deposition followed by low‐temperature scanning tunneling microscopy (STM) to decipher the self‐assembly, rearrangement, and disassembly processes of {Cr_6_}*_n_* horseshoe oligomers (*n* = 1–5). Activated tandem IM‐MS reveals the oligomer disassembly in detail, highlighting the stability of the dimer unit. When *n* = 2 and *n* = 3 oligomers are deposited on surfaces, we observe the rearrangement of dimers and trimers to dimers of dimers, and at higher coverages, the formation of an unexpected hexagonal‐like network. In its entirety, the experimental and computational data provide a convincing framework for the analysis of supramolecular assembly processes in noncrystalline phases that could be used in future design strategies.

## Graphical abstract

Using a combination of ion mobility mass spectrometry (IM‐MS), tandem mass spectrometry (MS^2^), and low‐temperature scanning tunneling microscopy (STM), we elucidate the self‐assembly, rearrangement, and disassembly processes of polymetallic {Cr_6_}*_x_* (*x* = 1 – 5) horseshoe oligomers.

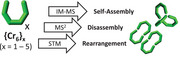

## Introduction

Molecular assembly is a key phenomenon in structural biology and supramolecular chemistry, relying on the enthalpically driven formation of intermolecular noncovalent interactions. This process is not only crucial for the formation of protein complexes,^[^ ^1^ ^]^ lipid membranes,^[^ ^2^ ^]^ biomolecular condensates,^[^ ^3^ ^]^ or DNA,^[^ ^4^ ^]^ enabling life on earth, but also for the design of novel molecules and materials. Despite broad interest, assembly processes are poorly understood and tools by which assembling intermediates can be investigated are lacking, which currently limits understanding and design efforts.^[^ ^5^, ^6^, ^7^ ^]^

Mass spectrometry (MS) is uniquely positioned to monitor assembly pathways and intermediates, for example, the aggregation of proteins to amyloid assemblies. Different gas‐phase techniques can be used to follow the conformations and stoichiometries of protein oligomers, contributing to our understanding of neurodegenerative diseases such as Alzheimer's and amyloidosis.^[^ ^8^, ^9^, ^10^, ^11^ ^]^

Mass spectrometry techniques are ideally suited to address problems in supramolecular chemistry, yielding information about stoichiometrically defined molecules in the absence of solvents or counter ions in the gas phase.^[^ ^12^, ^13^, ^14^, ^15^, ^16^, ^17^ ^]^ Advancements in MS have led to the direct structural characterization of ions in the gas phase, for example, with ion mobility mass spectrometry (IM‐MS) experiments in which structure and mass are measured in the same experiment.^[^ ^18^ ^]^ Structural information are provided as collision cross sections (CCS), which correspond to size and shape of a given ion and can be compared to values obtained from candidate geometries.^[^ ^17^, ^18^ ^]^ Although IM‐MS is more commonly applied for biological systems, it has become increasingly popular in the field of supramolecular chemistry^[^ ^17^, ^19^, ^20^ ^]^ and coordination‐driven self‐assembly.^[^ ^13^, ^21^ ^]^

In previous work, we used IM‐MS to investigate a series of polymetallic rings and rotaxanes, demonstrating how their stability and disassembly can be tuned by varying the metal composition, carboxylate ligands and rotaxane stopper groups.^[^ ^22^, ^23^, ^24^ ^]^ We also showed how the use of different adduct ions can inform about structural trends of these and similar complexes,^[^ ^25^ ^]^ and revealed a pathway for the formation and characterization of smaller polymetallic rings in vacuo, based on collisional activation of larger precursors.^[^ ^26^, ^27^ ^]^

Complementary to self‐assembly, molecular disassembly can be investigated using tandem mass spectrometry (MS^2^).^[^ ^28^ ^]^ Commonly, this involves *m*/*z*‐selection of ions and subsequent activation with an inert gas at user‐defined energies. Such collisions induce structural changes, which are often accompanied by fragmentation. When coupling MS^2^ to IM‐MS, this can be monitored by measurements of CCS and *m*/*z*.^[^ ^29^ ^]^

The combined use of electrospray ionization beam deposition (ESIBD) and low‐temperature scanning tunneling microscopy (STM) has emerged as an elegant way to investigate gas‐phase structures at a single‐molecule level with sub‐nm resolution.^[^ ^30^ ^]^ The ability to soft‐land ions from the gas phase onto a surface, via ESIBD, often enables the preservation of the intact structure of molecules as they are adsorbed onto a surface from the gas phase.^[^ ^31^ ^]^ Subsequent imaging post‐deposition using STM allowed characterization of complex biomolecules such as glycans^[^ ^32^, ^33^, ^34^ ^]^ and glycoconjugates,^[^ ^35^ ^]^ peptides,^[^ ^36^, ^37^ ^]^ as well as polymetallic rings.^[^ ^38^ ^]^ ESIBD has also been used to study the self‐assembly of peptides^[^ ^39^ ^]^ and nanoribbons,^[^ ^40^, ^41^ ^]^ enabling detailed understanding of how such molecules assemble on surfaces.

In this study, we investigate the assembly processes of polymetallic Cr*_x_* horseshoes, which occur in a dynamic solution equilibrium of ions with the formula {Cr*_x_* F*_x_*_+5_(O_2_C*^t^* Bu)_2_ *_x_*_‐2_}^3−^.^[^ ^42^, ^43^, ^44^ ^]^ These horseshoes are finite chromium chains with free fluoride ligands, which coordinate charge‐balancing secondary ammonium cations [NH_2_RR’]^+^ via hydrogen bonds in the crystalline state. Through their terminal centers, such horseshoes can act as ligands and reactants for new polymetallic complexes in a manner that is dependent on *x* as well as R and R’, as demonstrated in the formation of a {Cr_6_}_4_CrF_6_Na_14_ compound.^[^ ^43^ ^]^ Cr*_x_* horseshoes have also been found to oligomerize, for example, in a {Cr_3_}*_n_* polymeric chain and a {Cr_6_}_4_ tetramer.^[^ ^43^ ^]^

We hypothesized that IM‐MS, STM, and MS^2^ could decipher similar assembly processes of polymetallic horseshoes, and for this study, we chose the horseshoe [NH_2_Et_2_]_3_[Cr_6_F_11_(O_2_C*^t^* Bu)_10_] = **M** (with *x* = 6). {Cr_7_M} rings have been proposed as qubits for quantum information processing,^[^ ^45^ ^]^ and complex supramolecules based on these qubits have been studied for quantum error correction.^[^ ^46^ ^]^ An open question is whether they can be deposited intact on surfaces, which is a necessary condition for them to be used in quantum devices. The horseshoe oligomers that we study here are closely related, but with *S* = 0 ground states and *S* = 1 low‐lying excited states,^[^ ^44^ ^]^ rather than *S* = ½ for {Cr_7_Ni}. The **M*_n_*** oligomers are particularly suitable for initial studies as the horseshoe shape is distinct and hence easily recognizable by STM.

We demonstrate the formation of a range of novel horseshoe oligomers **M*_n_*** (*n* = 1–5) and show how IM‐MS and STM, supported by density functional theory (DFT) calculations, can investigate the oligomers’ self‐assembly, rearrangement, and disassembly processes in detail, revealing fundamental trends in their stoichiometries, stabilities, and conformational landscapes. Experimental details can be found in the Supporting Information.

## Results and Discussion

**Figure 1:**
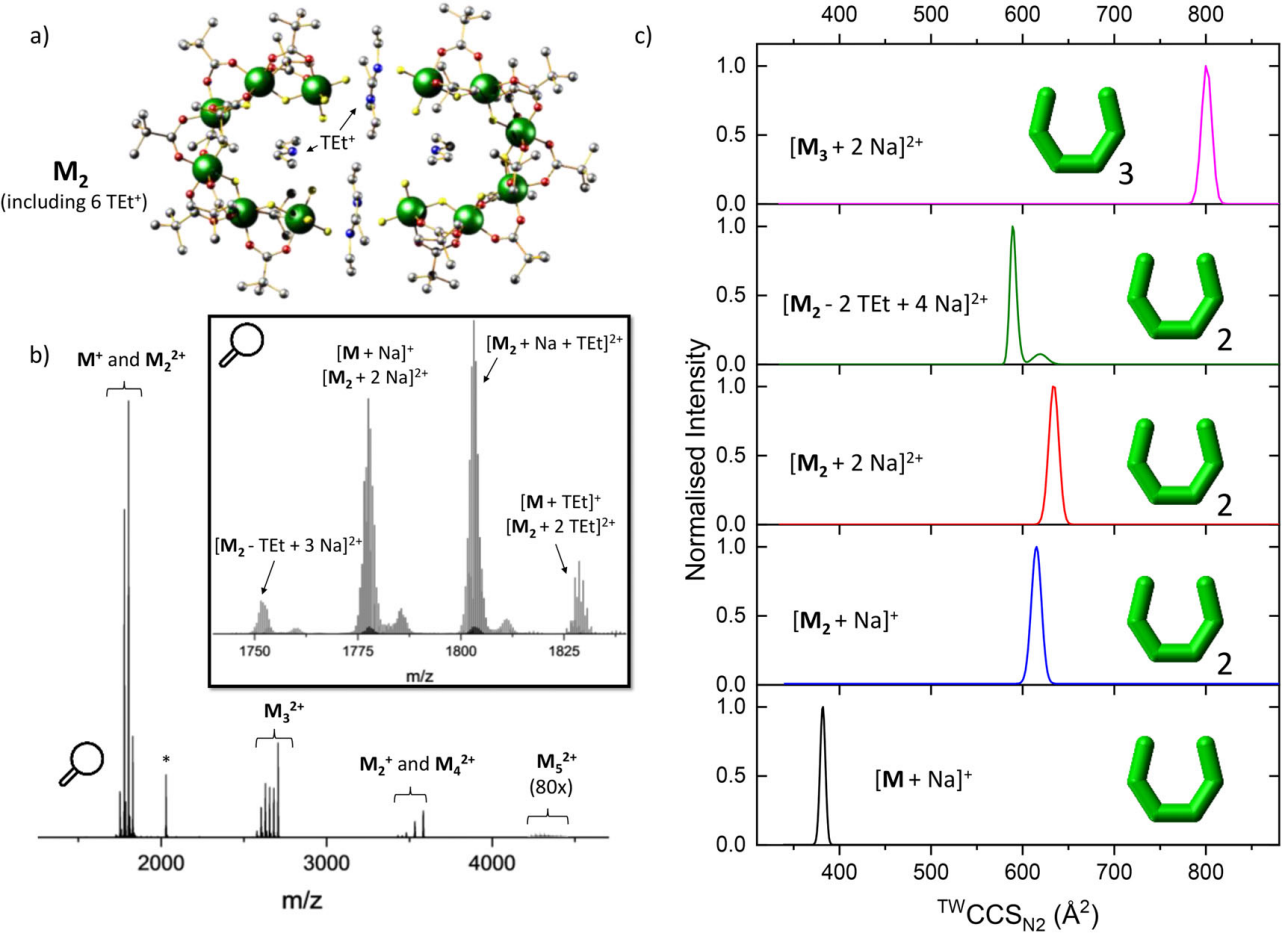
a) Crystal structure of the dimer **M_2_** (Cr: green, F: yellow, O: red, C: grey, and N: blue). Hydrogen atoms are omitted for clarity. b) Mass spectrum of **M** in NaI with three main ion regions: **M^+^**/**M_2_^2+^**, **M_3_^2+^**, and **M_2_^+^**/**M_4_^2+^**. The *m*/*z* of **M_5_^2+^** ions is zoomed in 80 times. *The ions at *m*/*z* = 2029 can be assigned as [**M** + 2 TEt + I]^+^. Inset: Zoom over the region between *m*/*z* = 1725 and 1850. Singly and doubly charged ions are labeled. c) ^TW^CCS_N2_ (TW = travelling wave) distribution of selected **M** cations.

The crystal structure of **M** presents as dimeric molecules **M_2_** via the noncovalent H─F bonds (Figure 1a), giving a first indication on self‐assembly preferences.^[^ ^42^ ^]^ **M** consists of six chromium (III) centers arranged in a semi‐open horseshoe structure, with each edge bridged by two pivalate ligands (O_2_C*^t^* Bu^−^ = Piv^−^) outside and one fluoride inside the ring. The two terminal Cr^III^ atoms are additionally coordinated by three fluoride ligands, which further exhibit hydrogen bonds to three secondary ammonium cations [NH_2_Et_2_]^+^ = TEt^+^, one in the center of **M** and two adjacent to the terminal fluorides.

### Self‐Assembly of Horseshoe Oligomers

A solution of **M** in NaI was transferred to the gas phase through nano‐electrospray ionization (nESI). The mass spectrum presents a series of cations with the general formula [**M*_n_*** – *k*TEt + (*k* + *z*) Na]*^z^* ^+^ (*n* = 1 – 5, *k* = (−2) – 8, *z* = 1 – 2), in which *n* represents the number of **M** monomers, *z* the charge state, and *k* informs about the number of Na^+^ and TEt^+^ cations present. This includes Na^+^ and/or TEt^+^ adducts (*k* = 0 – (−2)) of the **M** oligomers, as well as ions in which TEt^+^ cations were exchanged for Na^+^ (*k* = 1 – 8). The observed signals mainly group into three regions of the displayed mass spectrum, depending on the global cluster type **M*_n_^z^* ^+^** (Figure 1b): **M^+^** and **M_2_^2+^** at *m*/*z* = 1670 – 1830 (Figure 1b inset), **M_3_^2+^** at *m*/*z* = 2550 – 2710 as well as **M_2_^+^** and **M_4_^2+^** at *m*/*z* = 3370 – 3590. **M_5_^2+^** ions were also found between *m*/*z* = 4200 and 4410 with low intensity (Figures 1b and S1). The distributions between the oligomeric types **M*_n_^z^* ^+^** and between ions with different *k* vary from sample to sample and day to day, suggesting a highly dynamic system. As a general trend, larger oligomers with higher *n* are preferred for systems with higher *k* (and *z*), likely due to the lower steric demand of Na^+^ compared to TEt^+^, facilitating self‐assembly (Supplementary Dataset).

In the mass spectrum of **M**, ions of the types **M^+^**/**M_2_^2+^** and **M_2_^+^**/**M_4_^2+^** often overlap as they share the same *m*/*z*. IM allows us to separate these clusters as higher charged ions traverse faster through the drift cell and arrive earlier, even though they have a higher mass (Figure S2 for *m*/*z* = 1778). We extracted the arrival time distributions (ATD) of all observed cations [**M*_n_*** – *m* TEt + (*m* + *z*) Na]*^z^* ^+^ and converted them to collision cross section distributions in nitrogen (CCS_N2_, Table S1). As a measure of size, CCS_N2_ significantly increases with the number of **M** monomers (*n*) but also with decreasing *k*, when more TEt^+^ cations are present (for a fixed *n*, Figure 1c). We also observed a slight increase from ions of the type **M_2_^+^** to **M_2_^2+^**.

**Figure 2:**
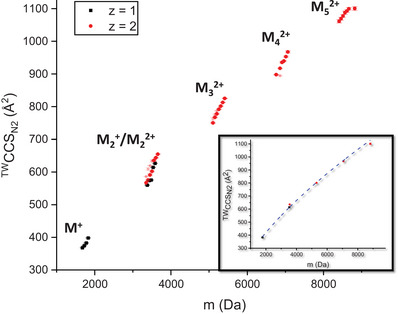
a) ^TW^CCS_N2_ of singly (*z* = 1, black) and doubly charged (*z* = 2, red) **M** cations versus their mass (Table S1). Minor conformations seen for M^2+^ are represented with smaller, red symbols. Error bars are shown and are smaller than the symbol size in some cases. Inset: Fit of the ^TW^CCS_N2_ data from the sodium adducted forms [**M*_n_*** + *z* Na]*^z^* ^+^ (*n* = 1 – 5, *z* = 1 – 2) with the function ^TW^CCS_N2_ = *A* · *m* ^2/3^, with A being a free variable. It cannot be completely excluded that directed growth occurs from monomer to dimer.

All **M*_n_*** ions with solely sodium charge carriers [**M*_n_*** + *z* Na]*^z^* ^+^ exhibit unimodal CCS_N2_ distributions; however, some ions of the type [**M_2_** – *k* TEt + (*k* + 2)  Na]^2+^ and less pronounced [**M_3_** – *k* TEt + (*k* + 2) Na]^2+^ appear with two conformations (Figures 1c and 2). One is dominant and has a smaller CCS_N2_, whereas the less abundant distribution appears at higher CCS_N2_. We attribute this to the exchange of TEt^+^ cations that are either intermolecularly (between the terminal Cr of different **M**) or intramolecularly bridging (between the terminal Cr of **M**, Figure 1a). We suggest that the smaller and dominant conformer is a result of intermolecular exchange, whereas the larger distribution corresponds to the exchange of an intramolecular TEt^+^ bridge. The smaller Na^+^ is presumably less suitable to intramolecularly bridge the fluorides than TEt^+^ due to the large diameter of **M**, and hence the exchange of the intramolecular TEt^+^ likely results in a larger conformer due to the absence of the bridge and subsequent expansion (see Disassembly of Horseshoe Oligomers). As the intramolecular bridge‐induced contraction remains for the exchange of intermolecularly bridging TEt^+^, we observe conformations with slightly smaller CCS_N2_. The higher abundance of the loss of TEt^+^ that is bridging intermolecularly (Figure 1c) can be explained stochastically (two intermolecular TEt^+^ vs. one intramolecular per **M**, Figure 1a) and with its easier accessibility.

The correlation between CCS_N2_ and the ion mass is a suitable tool to assess the packing density of synthetic molecules and to assess the structural landscape of oligomers.^[^ ^17^, ^26^, ^47^ ^]^ CCS_N2_ of the **M*_n_*** clusters follows the relationship CCS_N2_ *= A · m* ^2/3^ (*A* = free variable, *m* = mass), which is an established correlation for undirected and 3D isotropic growth (Figure 2).^[^ ^8^ ^]^ Both the charge state (*z*) and in particular the number of TEt^+^ and Na^+^ (*k*) influence the CCS_N2_ strongly, and therefore the fit was performed exclusively with the pure sodium adducts [**M*_n_*** + *z* Na]*^z^* ^+^ (*n* = 1 – 5, *z* = 1 – 2; Figure 2 inset). DFT was used to obtain optimized structures for [**M** + Na]^+^ and [**M_2_** + Na]^+^ (Figure S3) starting from the published dimeric crystal structure (and by adding Na^+^, as discussed in Disassembly of Horseshoe Oligomers). Theoretical CCS_N2_ for both ions were predicted using the trajectory method of IMoS on the DFT‐optimized structures, yielding CCS_N2_ of (407.3 ± 1.0) Å^2^ for [**M** + Na]^+^ and (659.1 ± 1.7) Å^2^ for [**M_2_** + Na]^+^, respectively. These values are ca. 7% higher than experimental data (Table S1), which is in the same order of magnitude as previously observed discrepancies between experimental and theoretical CCS_N2_ of synthetic molecules^[^ ^48^ ^]^ and specifically polymetallic complexes of the same family.^[^ ^22^, ^25^, ^26^ ^]^

### Rearrangement of Horseshoe Oligomers

**Figure 3:**
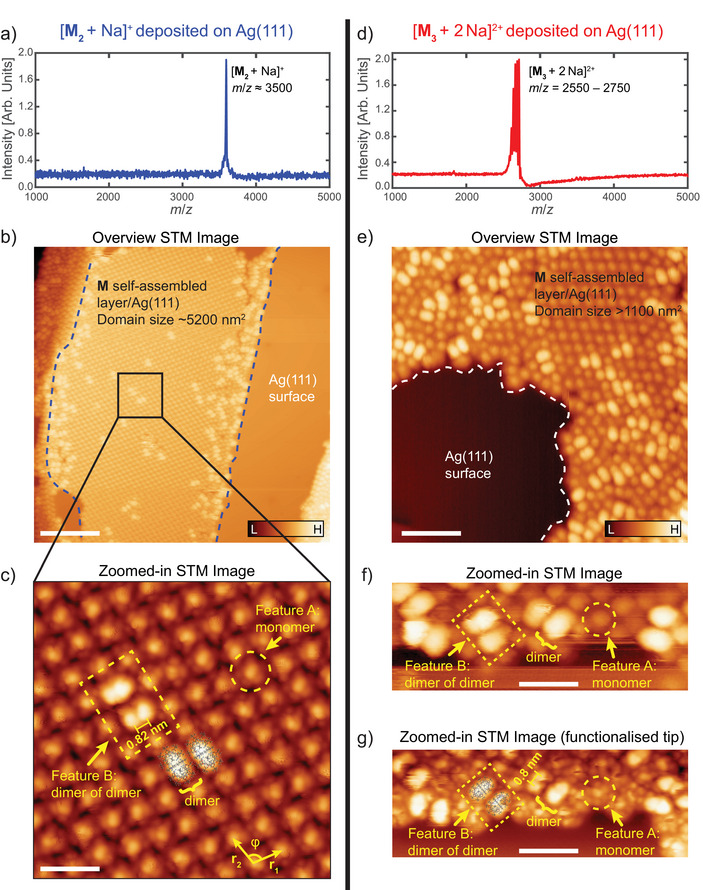
a) and d) Mass spectrum after *m*/*z*‐selection of [**M_2_** + Na]^+^ (a, *m*/*z* = 3532) and [**M_3_** + 2 Na]^2+^ (d, *m*/*z* = 2655) for deposition onto an Ag(111) surface. b) and e) Constant‐current STM image, with tunneling set point of *I*_t_ = 5 pA, *V*_b_ = 300 mV, of an Ag(111) surface held at room temperature after exposure to the *m*/*z*‐selected ion beam. STM images (11 K) show a large 2D ordered (b and e) domain comprised of reassembled **M*_x_*** molecules on the Ag(111) surface, respectively. c) and f) A zoomed‐in constant‐current STM image, with tunneling setpoint of *I*_t_ = 5 pA, *V*_b_ = 300 mV of the ordered domain (b, black box) and a sub‐monolayer domain edge (f). The ordered (b – g) sub‐monolayers consist of molecular feature **A**, identified as **M** monomers, self‐assembling to form a 2D structure with lattice vectors of lengths |*r*_1_| = 1.72 ± 0.06 nm and |*r*_2_| = 1.71 ± 0.05 nm separated by an angle of φ = 107 ± 1°. The brighter feature **B** seen corresponds to **M_4_** dimers of dimers located in the **M** monolayer. g) STM imaging of the same area as in (f) but with a functionalized tip, enhancing the features observed in STM imaging in (f). Scale bars: 20 nm (Figure 3b), 3 nm (Figure 3c), 8 nm (Figure 3e), 4 nm (Figure 3f,g).

To examine how the horseshoe dimer **M_2_** contributes to higher‐order assemblies, we selected ions of the type [**M_2_** + Na]^+^ in a quadrupole mass filter at *m*/*z* = 3532 (Figure 3a). These ions were deposited on a noble Ag(111) metal substrate held at room temperature and with a retarding voltage applied to the surface, ensuring soft‐landing conditions. The surface was subsequently imaged with STM at cryogenic temperatures (11 K, Figure 3b,c).

On the Ag(111) surface, we found that the deposited molecules readily form large self‐assembled domains with patches of the clean surface still visible. These domains appear highly ordered (Figure 3b, blue line), implying that units from the *m*/*z* selected [**M_2_ **+ Na]^+^ are highly mobile on the Ag(111) surface. This was confirmed by STM tip manipulation (Figure S4).

A closer examination of the ordered domains after [**M_2_** + Na]^+^ deposition reveals two distinct molecular features **A** and **B** (Figure 3c). **A** (dashed circle) forms the 2D ordered sub‐monolayer on the Ag(111) surface. These molecular features are ≈1.7 nm apart in both dimensions of the monolayer (Table S2), which is too small for a **M_2_** dimer (≈2.3 nm in length, Figure S3), but corresponds well to the dimensions of the **M** monomer (≈1.6 nm in length; Figure S3). We therefore assign **A** to correspond to the **M** monomer. We note that the total size of two adjacent **A**’s is too large for a **M_2_** dimer, suggesting that the sub‐monolayer is comprised of **M** molecules dissociated from their **M_2_** parent structure upon deposition. These **M** monomers are then free to diffuse and self‐assemble to form the ordered layer observed in STM imaging. The second feature, **B** (Figure 3c, yellow dashed box), is much larger and appears brighter than the **A** monomers. Unexpectedly, STM tip manipulation revealed that these features lie within the same monolayer as the **A** monomers (Figure S5).

**B** consists of two building blocks (Figure 3c, yellow bracket) that have dimensions that are more elongated than **A** and show two maxima with a separation of ≈0.82 nm (yellow solid line). These dimensions coincide well with a **M_2_** dimer structure (molecular structure overlaid in Figures 3c and S3). Therefore, we can assign **B**, comprising a pairing of **M_2_** dimers, to be **M_4_** dimers of dimers. The dimensions suggest a flat structure of **M_4_** on the surface, which is in contrast to a previously published tetrameric horseshoe crystal structure.^[^ ^43^ ^]^

We note that we do not observe these **M_4_** dimers of dimers on the bare Ag(111) surface but only in the **M** sub‐monolayer. This suggests surface‐driven disassembly upon direct Ag(111) surface adsorption. Surprisingly, the vast majority of the **M_2_** dimers in the **M** monolayer are found in pairs forming the **M_4_** dimers of dimers (yellow dashed box in Figure 3c). One possible explanation is that the diffusion barrier for a sole **M_2_** dimer on a clean Ag(111) surface is made even larger when **M_2_** dimers are adjacent to one another, implying dimer–dimer stabilizing interactions.^[^ ^49^ ^]^ This is supported by STM tip manipulation, which showed that the removal of the entire **M_4_** dimer of dimers, even though only the **M_2_** dimer was targeted via tip manipulation (Figure S5). The assembly from monomers to a stable dimer of dimers is also supported by the observation that the dimensions for **B** are not the same as for the underlying monolayer. The distance between the monomers in each dimer subunit is ∼0.8 nm and between the two dimers is ∼1.7 nm.

The ion beam selecting at *m*/*z* = 3532 contains a mixture of [**M_2_** + Na]^+^ and [**M_4_** + 2 Na]^2+^ (Figure 1b); however the former is the dominant feature. To verify that the **M_4_** structures found on Ag(111) are the product of rearrangements on the surface and not of gas‐phase deposition, we repeated the same deposition and imaging workflow for [**M_3_** + 2 Na]^2+^ (Figure 3d–g), where the mass spectrum shows no overlap with other oligomer sizes (Figure 1b).

**Figure 4:**
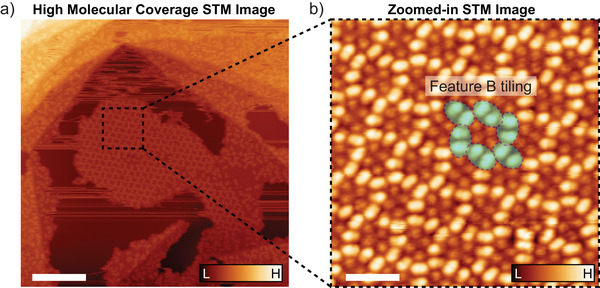
Left: Constant‐current STM image, with tunneling set point of *I*_t_ = 0.1 pA, *V*_b_ = 300 mV, of an Ag(111) surface held at room temperature after exposure to the *m*/*z*‐selected ion beam of [**M_3_** + 2 Na]^2+^ (*m*/*z* = 2655). The surface was exposed to the ion beam long enough for a high molecular coverage on the surface. Right: Constant‐current STM imaging of the area indicated in a). We find that the **M_4_** dimers of dimers (feature **B**, indicated in cyan) assemble to a mostly pseudo‐hexagonal structure in the high‐molecular coverage limit. Scale bars: 40 nm (left), 6 nm (right).

We observe similar 2D self‐assembled domains (Figure 3e). Higher magnification shows features that are like those observed following [**M_2_** + Na]^+^ deposition; **A** (yellow dashed circle in Figure 3f) and **B** (yellow dashed box in Figure 3f). Comparison with the features observed for [**M_2_** + Na]^+^ yielded highly comparable dimensions (Table S2), supporting their identities. Tip‐enhanced resolution confirmed two dimer units forming **B**, and hence **B**’s assignment as **M_4_** dimers of dimers (Figure 3g). The observation of predominantly **M_2_**/**M_4_** in the STM images of deposited **M_3_** also supports the hypothesis of rearrangement during or after deposition, leading to a **M_4_** dimer of dimer on the surface consisting of two **M_2_** dimers. We note that in the case of deposition of [**M_3_** + 2 Na]^2+^ on Ag(111), the resulting self‐assembled domains are not as ordered as those after depositing [**M_2_** + Na]^+^, and we suggest this is a result of competing kinetic and thermodynamic factors. A plausible scenario is that the adsorbed molecules interact in different geometries that all have similar interaction energies due to the dominance of van der Waals interactions. For example, in the case of two **M** interacting dynamically, the **M_2_** dimer may not have reached the most stable geometry at the time a third **M** arrives to interact. This can lead to the trimeric **M_3_** being trapped in an unfavorable geometry, resulting in a disordered assembly as in the case of [**M_3_** + 2 Na]^2+^ on Ag(111). Alternatively, when the dimer reaches its lowest energy geometry prior to the arrival of the third **M**, an ordered region could form as in the case of [**M_2_** + Na]^+^ on Ag(111). Interestingly, at even higher coverages, we observed a striking pseudo‐hexagonal assembly of **M_4_** dimer of dimers on Ag(111) (Figure 4). Particularly unusual is the irregularity of the array, in which most, but not all, hexagons consist of 5 features **B** (4 × features **B** + 2 × features **B** halves = 20 monomers).

Here, Ag(111) substrates were predominantly chosen for their inertness and are known to facilitate the self‐assembly of various supramolecules.^[^ ^49^ ^]^ We propose that the self‐assembly of the molecules on this surface is primarily driven by van der Waals interactions between the pivalate ligands of neighboring **M** unit; however, due to the bulky and nonplanar nature of **M**, steric effects likely also play a role in dictating the packing geometry. As the Ag(111) substrate only weakly interacts via nonspecific van der Waals interactions, the substrate does not impose a strong templating effect and allows the **M** units to remain mobile and explore different intermolecular configurations on the surface. Thus, the final assembly is primarily determined by the balance of intermolecular dispersion forces and steric packing considerations between the **M** units. To elucidate this effect further, we have also explored the self‐assembly of **M** on the more reactive substrate Cu(100) after deposition of [**M_2_** + Na]^+^ and [**M_3_** + 2 Na]^2+^, showing that although rearrangements occur to **M** and **M_2_**, no **M_4_** dimer of dimers is found (Figures S6 and S7). This agrees with the higher diffusion barrier for **M** on Cu(100) compared to Ag(111), limiting the surface‐driven assembly.

### Disassembly of Horseshoe Oligomers

Ions of the type [**M*_n_*** + *z* Na]^z+^ (*n* = 1 – 5, *z* = 1, 2) were subjected to activated tandem ion‐mobility mass spectrometry. Target ions were isolated and collisionally activated at user‐defined kinetic energies, which led to fragmentation. The ATD was simultaneously measured for both precursor and fragment ions.

The disassembly of the higher order horseshoe oligomers **M*_n_*** (*n* = 3 – 5) shows the retention of the dimeric unit in all cases. For the trimer, this involves predominantly the disassembly of the trimer via **M_3_** → **M **+** M_2_** (Figure S8a), whereas the pentamer primarily dissociates via **M_5_** → **M_2_** +** M_3_** (Figure S8b). The disassembly of the tetramer proceeds via the homolytic fragmentation pathway **M_4_** → 2 **M_2_** (Figure S9). For the fragmentation of the odd‐numbered clusters, asymmetric distributions of Na^+^ and TEt^+^ ions were observed between **M_3_**/**M** and **M_2_** units, respectively, showing a strong preference for Na^+^‐rich dimers and TEt^+^‐rich monomers/trimers (Figure S8). This suggests intermolecular cation exchange, which is possibly driven by the preference of Na^+^ in **M_2_** over TEt^+^ due to steric repulsions between adjacent TEt^+^ ions.

**M_2_** units are more stable and follow two different pathways, with the preference depending on the charge state *z* of the precursor cluster **M_2_^z+^**. Singly charged dimers **M_2_^+^** predominantly lose several TEtF units before they fragment to **M^+^** monomers at higher energies (Figure S10). Conversely, the doubly charged species **M_2_^2^** ^+^ dissociate to **M^+^** ions and lose TEtF and NaF simultaneously (Figure S11).

We also examined the disassembly of the monomeric ion [**M** + Na]^+^, which is found to be less stable than its dimer. This species fragments via losses of TEtF, TEtPiv, and the amine [TEt – H] = NHEt_2_, but does not readily lose NaF or NaPiv (Figure S12). This is likely due to the lack of stabilizing hydrogen bonds of TEt^+^ (present in the dimer), which makes the TEt^+^ dissociation more likely than for Na^+^.

Tandem ion mobility (IMS^2^) tandem mass spectrometry allows us to map the conformational landscape of the horseshoe oligomer disassembly process. This was particularly useful for the dimeric ion [**M_2_** + 2 Na]^2+^, which was observed to break into **M^+^** ions involving concomitant losses of TEtF and/or NaF (Figure S11). The CCS_N2_ values of the fragment ions of [**M_2_** + 2 Na]^2+^ were enumerated and show similar results, suggesting that the structure of **M** (or **A**) is maintained upon the dissociation of **M_2_**. This agrees with the STM imaging results following deposition on Ag(111), where initially the dissociation of **M_2_** leads to an **M** monolayer (Figure 3c).

**Figure 5:**
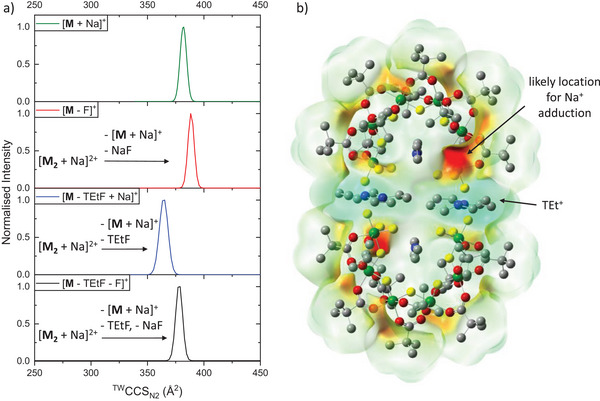
a) ^TW^CCS_N2_ distribution of [**M** + Na]^+^ (as in Figure 1c) and of three selected fragments obtained from collisional activation of [**M_2_** + 2 Na]^2+^. For the ^TW^CCS_N2_ distributions of the fragments, [**M_2_** + 2 Na]^2+^ was isolated with a quadrupole at *m*/*z* = 1778 and selected by ion mobility (IMS^2^), as well as activated via collisions at *E*_lab_ = 100 eV. The data shows a CCS_N2_ increase for loss of NaF and CCS_N2_ decrease for loss of TEtF, which we attribute to conformational differences. An alternative, although less likely explanation, is differences in the electronic interactions of the polarizable N_2_ gas with different charge carriers. b) Electrostatic potentials of **M_2_** mapped onto an electron density isosurface at 0.0004 au with ESP red −0.006 to blue +0.006 au (Cr: green, F: yellow, C: grey, O: red, N: blue, and H: omitted for clarity). The region of the bridging F^−^ next to the terminal Cr center is most negative; however, due to differences in Cr–F angles, this is not the case for the other terminal Cr on the same horseshoe unit. This site and the analogous region in the back of the other **M** unit are the most likely locations for Na^+^ adduction.

Interestingly, subtle conformational differences were observed in the fragments of [**M_2_** + 2 Na]^2+^. While TEtF losses lead to a CCS_N2_ decrease, an increase was observed when NaF is lost (Figure 5a), when compared to [**M** + Na]^+^ (likely **A**). We attribute this behavior to different fluorides involved in each fragmentation channel. For the TEtF leaving group, a terminal fluoride is likely lost along with the adjacent bulky TEt^+^, leading to a more compact structure with a correspondingly smaller CCS_N2_. Conversely, we suggest one of the bridging fluorides to be involved in the loss of NaF, which would result in the cleavage of a fluoride bridge and hence rearrangement to a more extended conformation. This can be explained with the Na^+^ addition adjacent to a bridging F^−^, agreeing well with the predicted electrostatic potential surface of **M_2_** obtained from DFT calculations (Figure 5b). The potential surface shows that the region of the bridging F^−^ adjacent to one terminal Cr center is most prone to bind the charge carrier Na^+^, and the subsequent loss of NaF at this position, and hence the loss of the F^−^ bridge, would result in a higher flexibility of the terminal Cr unit, an extension of the structure and therefore a higher CCS_N2_.

Taken together, the data from tandem ion mobility tandem mass spectrometry measurements suggest that the dominant fragments are due to rupture at the interface of the crystallographic dimer, which supports the hypothesis that this **M_2_** structure is stable in the gas phase. Utilizing such accessible stoichiometric and conformational changes could aid the manipulation of assembly processes in the future, for example, when depositing the observed fragment ions (Figure 5a) on surfaces. The obtained ions are similar to feature **A**; however, the geometry and composition of their opening are changed. This could lead to different assembly routes and provide an informed route for systematic exploitation of **M** superstructures.

### Discussion on Assembly Processes

**Figure 6:**
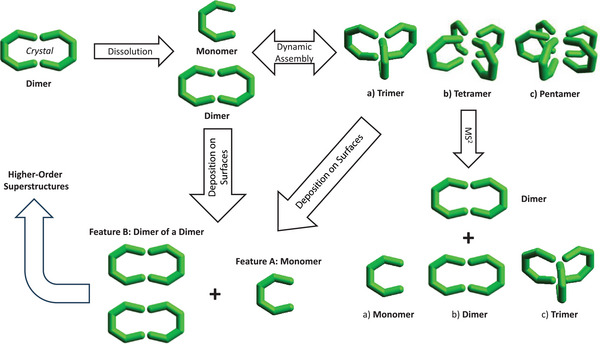
Summary of the self‐assembly, rearrangement, and disassembly pathways of M*_x_* oligomers in solution (top), in the gas phase (bottom right), and on surfaces (bottom left). Schematic structures are suggested based on IM‐MS, DFT, MS^2^, and STM data.

The path of {Cr_6_}*_n_* oligomerization likely proceeds via two routes: dissociation of the **M_2_** crystal structure (Figure 1a) to **M**, which then allows the formation of odd‐numbered oligomers **M*_x_*** (*x* = 3, 5) (Figure 6). Second, oligomerization could also proceed through dimerization of **M_2_**, yielding a tetramer. We have previously crystallized tetramers involving dimethylammonium counterions, in which the terminal Cr1 and Cr6 atoms of each horseshoe lie in the same single planes, respectively, with the horseshoe planes themselves being perpendicular to the center square (Figure 6 top right).^[^ ^42^ ^]^

The oligomers’ structures can be investigated using IM‐MS, suggesting an overall isotropic growth of the oligomers in three dimensions (Figure 2). This excludes a directed 2D growth that would be comparable to clover leaves. The oligomer disassembly, as probed by MS^2^, revealed stable dimers **M_2_** that dissociate to monomers at high collision energies. The most likely explanation for the high dimer stability is a structure similar to that of the **M_2_** crystal structure (Figure 1a), further supported by DFT calculations and MS^2^ data. Tetramers are found to readily dissociate into two dimers, suggesting these assemblies are weakly bound.

Pentamers disassemble to trimers and dimers, and trimers to dimers and monomers, suggesting weakly bound **M** units attached to dimers in **M_3_** and **M_5_**. The chemically most likely structures are those similar to the above‐described tetramer (Figure 6), and this agrees well with the 3D growth suggested by IM‐MS measurements.

Probing the structure of dimers and trimers via ESIBD and STM revealed a fascinating rearrangement process, which involves the dissociation of both species to monomers (**M_2_** units are possibly partially retained) and rearrangement to a polymeric layer involving **A**/**M**. In the same layer, units of **B**/**M_4_** appear in the shape of two bound dimers flat on the surface, unlike the suggested tetramer structure in the gas phase. At higher coverages, a honeycomb structure dominates (Figure 4).

An interesting route to guide assembly processes of such horseshoes could be the manipulation of their building blocks by MS^2^ in the gas phase (Figure 5), prior to deposition. The observed subtle changes in composition and conformation upon fragmentation, occurring at the interface of the **M_2_** unit, possibly lead to different assembly stoichiometries and structures, further contributing to the exploitation of self‐assembly for synthetic chemistry.

The combination of IM‐MS, MS^2^, DFT, and STM provides a complementary picture of self‐assembly, rearrangement, and disassembly preferences and structures of {Cr_6_}*_n_* horseshoe oligomers, from solution to the gas phase and then onto surfaces, opening new routes toward the utilization of self‐assembly for metallosupramolecular oligomers.

## Conclusion

We demonstrated that IM‐MS, MS^2^, and low‐temperature STM are highly suitable tools to follow the self‐assembly, rearrangement, and disassembly of {Cr_6_}*_n_* horseshoe oligomers. The data presented suggests dynamic assembly in solution with an isotropic oligomer growth from monomer to pentamer and a stability preference for the dimer unit M_2_. Conformational diversity in both the oligomers and their fragments was revealed with IM‐MS and supported by DFT calculations, providing further insights into their specific binding preferences and disassembly trends, which in turn could guide future synthetic chemistry in noncrystalline phases. On surfaces, we found an unexpected rearrangement mechanism from M_2_ and M_3_ to a polymeric layer with monomers and dimers of dimers, before at higher coverages a hexagonal‐like network dominates. Taken together, the experimental and computational data establish a framework for the design, analysis, and future exploitation of molecular assembly processes of supramolecules.

## Supporting Information

The authors have cited additional references within the Supporting Information.^[^ ^50^, ^51^, ^52^, ^53^, ^54^, ^55^, ^56^, ^57^, ^58^, ^59^, ^60^, ^61^ ^]^

## Conflict of Interests

The authors declare no conflict of interest.

## Supporting information



Supporting Information

## Data Availability

A supplementary dataset is available on Figshare (https://doi.org/10.6084/m9.figshare.27255912.v3), containing the raw data of ion mobility mass spectrometry and mass spectrometry measurements, STM images as well as the outputs from DFT calculations.
