# Supplementary material for: Self‐Assembly, Rearrangement, and Disassembly of {Cr6} Horseshoe Oligomers
Source: Angew Chem Int Ed Engl. 2025 Oct 5;64(49):e202510610. doi: 10.1002/anie.202510610 (PMC12668306; doi:10.1002/anie.202510610)
Supplement: Supplementary file 1 — Supporting Information [file ANIE-64-e202510610-s001.docx]

**Self-Assembly, Rearrangement and Disassembly
of {Cr_6_} Horseshoe Oligomers**

Niklas Geue^1,✝,*^, Dhaneesh Kumar^2,✝^, Jimin Ham^2^, Shengpeng Huang^2^,
Grigore A. Timco^3^, Neil A. Burton^3^, Richard E. P. Winpenny^3,*^,
Kelvin Anggara^2,*^ and Perdita E. Barran^1,*^

*^1^Michael Barber Centre for Collaborative Mass Spectrometry, Manchester Institute of Biotechnology, Department of Chemistry, The University of Manchester, 131 Princess Street, Manchester, M1 7DN, UK. ^2^Nanoscale Science Department, Max Planck Institute for Solid State Research, Heisenbergstr. 1, D-70569 Stuttgart, Germany. ^3^Department of Chemistry, The University of Manchester, Oxford Road, Manchester, M13 9PL, UK.*

^✝^ These authors contributed equally.

Corresponding authors: niklas.geue@manchester.ac.uk, richard.winpenny@manchester.ac.uk, k.anggara@fkf.mpg.de, perdita.barran@manchester.ac.uk

Table of Contents

[**Methods** 3](#_Toc195712402)

[**Figure S1:** Zoom in the mass spectrum of **M** for *m/z* = 4200 – 4400. 6](#_Toc195712403)

[**Figure S2:** Mass spectrum and arrival time distribution of [**M** + Na]^+^ and [**M_2_** + 2 Na]^2+^. 7](#_Toc195712404)

[**Table S1:** *^TW^CCS_N2_* values of the ions [**M_n_** – k TEt + (k + z) Na]^z+^ 8](#_Toc195712405)

[**Figure S3:** DFT optimised structures of a) [**M** + Na]^+^ and b) [**M_2_** + Na]^+^ 10](#_Toc195712406)

[**Figure S4:** STM image of [**M_2_** + Na]^+^ on Ag(111) after STM tip manipulation. 11](#_Toc195712407)

[**Table S2:** Measurement of features **A** and **B** as observed in Figure 3. 12](#_Toc195712408)

[**Figure S5:** STM tip manipulation of **M_4_**. 13](#_Toc195712409)

[**Figure S6:** STM images for the deposition on Cu(100). 14](#_Toc195712410)

[**Figure S7:** STM image for the deposition of [**M_3_** + 2 Na]^2+^ on Cu(100). 15](#_Toc195712411)

[**Figure S8:** MS^2^ spectra of a) [**M_3_** + 2 Na]^2+^ and b) [**M_5_** + 2 Na]^2+^ 16](#_Toc195712412)

[**Figure S9:** ATDs of the ions [**M_2_** + Na]^+^ and [**M_4_** + 2 Na]^2+^ 17](#_Toc195712413)

[**Figure S10:** MS^2^ spectra of [**M_2_** + Na]^+^ at *E_lab_* = 110 eV and *E_lab_* = 140 eV 19](#_Toc195712414)

[**Figure S11:** MS^2^ spectra of [**M_2_** + 2 Na]^2+^ at *E_lab_* = 130 eV and *E_lab_* = 240 eV 20](#_Toc195712415)

[**Figure S12:** MS^2^ spectra of [**M** + Na]^+^ at *E_lab_* = 65 eV and *E_lab_* = 130 eV 21](#_Toc195712416)

[**Figure S13:** Mass spectra of **M** at concentrations = 10 – 100 µM. 22](#_Toc195712417)

# **Methods**

**Synthesis, Sample Preparation and Materials**

The horseshoe **M** was prepared according to a previously published route.^[1]^ **M** was typically transferred to the gas phase from 200 or 500 µM solutions of **M** in 500 µM NaI and 4:1 toluene/methanol. Lower sample concentrations did not predominantly yield intact **M** ions, which we attribute to an entropic threshold associated with the self-assembly of the monomeric **M** (Figure S13). All reagents and solvents were purchased from Sigma-Aldrich, Alfa, Fisher Scientific or Fluorochem and used without further purification.

**Ion-Mobility Mass Spectrometry (IM-MS) and Data Processing**

The sample solution was ionized with a nanoESI source and sprayed from borosilicate glass capillaries (World Precision Instruments, Stevenage, UK), which were pulled on a Flaming/Brown P-2000 laser puller (Sutter Instrument Company, Novato, CA, US). A potential of 1.0 - 1.5 kV was applied through a platinum wire (Diameter 0.125 mm, Goodfellow, Huntingdon, UK) inserted into the nanoESI capillaries. The source temperature was 23 ºC.

Ion mobility mass spectrometry (IM-MS) experiments were performed on a Select Series Cyclic IMS (Waters).^[2]^ After the transfer to the gas phase (Cone Voltage: 20 V, Source Offset: 10 – 30 V, Purge Gas: 0 – 300 L/h), ions of interest were isolated by a quadrupole mass filter, activated in a trap cell at user-defined energies where appropriate (Trap Bias: 2 V, Voltage: 0 – 200 V) and subsequently injected into the cyclic ion mobility drift ring. In this region, ions were separated by using a non-uniform electric field under a constant nitrogen pressure. Travelling waves (TW, Height: 20 – 22 V) pushed the ions through the cyclic drift region. Unless noted otherwise, ions travelled one pass in the cyclic drift ring (“single path”, separation time: 2 – 27 ms) and were subsequently transferred (Transfer Energy: 0 – 15 V) to a time-of-flight mass analyser. For tandem IM-MS experiments, ions of interest were mobility-selected (IMS^1^) and stored in the pre-array store, and subsequently reinjected into the array and the cyclic ion mobility cell (IMS^2^). Collisional activation at user-defined energies was applied if appropriate. Details of travelling-wave ion mobility spectrometry (TWIMS)^[3–5]^ and the instrument design^[2]^ can be found elsewhere. More detailed experimental parameters can be found in the raw data deposited in the Supplementary Dataset.

Experimentally obtained arrival time distributions were converted to nitrogen collisional cross sections (*^TW^CCS_N2_,* TW = “Travelling Waves”) *via* published calibration procedures.^[6]^ The Agilent tune mix was used for all *^TW^CCS_N2_* calibrations.^[7]^

**Density Functional Theory and Collision Cross Section Calculations**

All DFT calculations were carried out with Gaussian 16^[8]^ utilizing the B3LYP exchange-correlation functional with the Grimme D3 empirical dispersion correction.^[9]^  An effective core potential and its associated split valence basis set were used for transition metals (LANL2DZ),^[10]^ and a 6-31G(d) basis set on other atoms.  All structures were optimized to the default convergence criteria (RMS force < 3 ∙ 10^-4^ E_h_/a_0_). Metal electronic states were high spin, as found experimentally, with low deviations from <S^2^>, although were ferromagnetically coupled. Atomic charges were obtained for the optimized structures at the same DFT level using the Merz-Kollman method with UFF based radii as implemented in Gaussian 16. An electrostatic potential surface (red negative to blue positive) of the geometry optimised horseshoe dimer **M_2_** computed using DFT (B3LYP/LAN2DZ-6-31G(d)/D3).

Theoretical *CCS_N2_* were obtained from the software IMoS by using the trajectory method in nitrogen gas including quadrupole potential (number of orientations 3, gas molecules per orientation 300,000, temperature 298 K, and pressure 101,325 Pa = 1 atm).^[11]^

**Ion Soft-Landing and Low Temperature Scanning Tunnelling Microscopy**

Ions were soft landed on an atomically flat Ag(111) (obtained by repeated cycles of Ar^+^ sputtering and annealing at 790 K) or a Cu(100) surface. The surfaces were held in ultrahigh vacuum (UHV) using a home-built electrospray ion beam deposition (ESIBD) apparatus at the Max Planck Institute for Solid State Research, Stuttgart, Germany. In the ESIBD workflow, described in detail elsewhere,^[12]^ molecular ions were generated by nanoESI using a metal-coated capillary tip, delivered to an inlet capillary (held at room temperature) and *m/z*-selected. *m/z*-selection was confirmed by characterizing the ion beam using the time-of-flight mass spectrometer in the ESIBD instrument (Figure 3a, d). The purified ion beam was aimed towards a clean Ag(111) or Cu(100) surface held at RT. By applying appropriate retarding bias to the surface, the landing energy of the ions was selected to be as low as possible (below ~10 meV/atom), which is well within the soft-landing regime (below ~40 meV/atom) that preserves the chemical structure of the landed ions.^[13,14]^ The base pressure during the ion beam deposition at the surface was <5 ×10^−10^ mbar.

The prepared surfaces with **M_x_** molecules were transferred to a low-temperature (11 K) scanning tunnelling microscope (Scienta Omicron Fermi SPM), where the surface and molecules were imaged under constant-current imaging conditions using a typical tunnelling current setpoint of 0.1 pA to 5 pA with a tunnelling bias of 0.3 V.


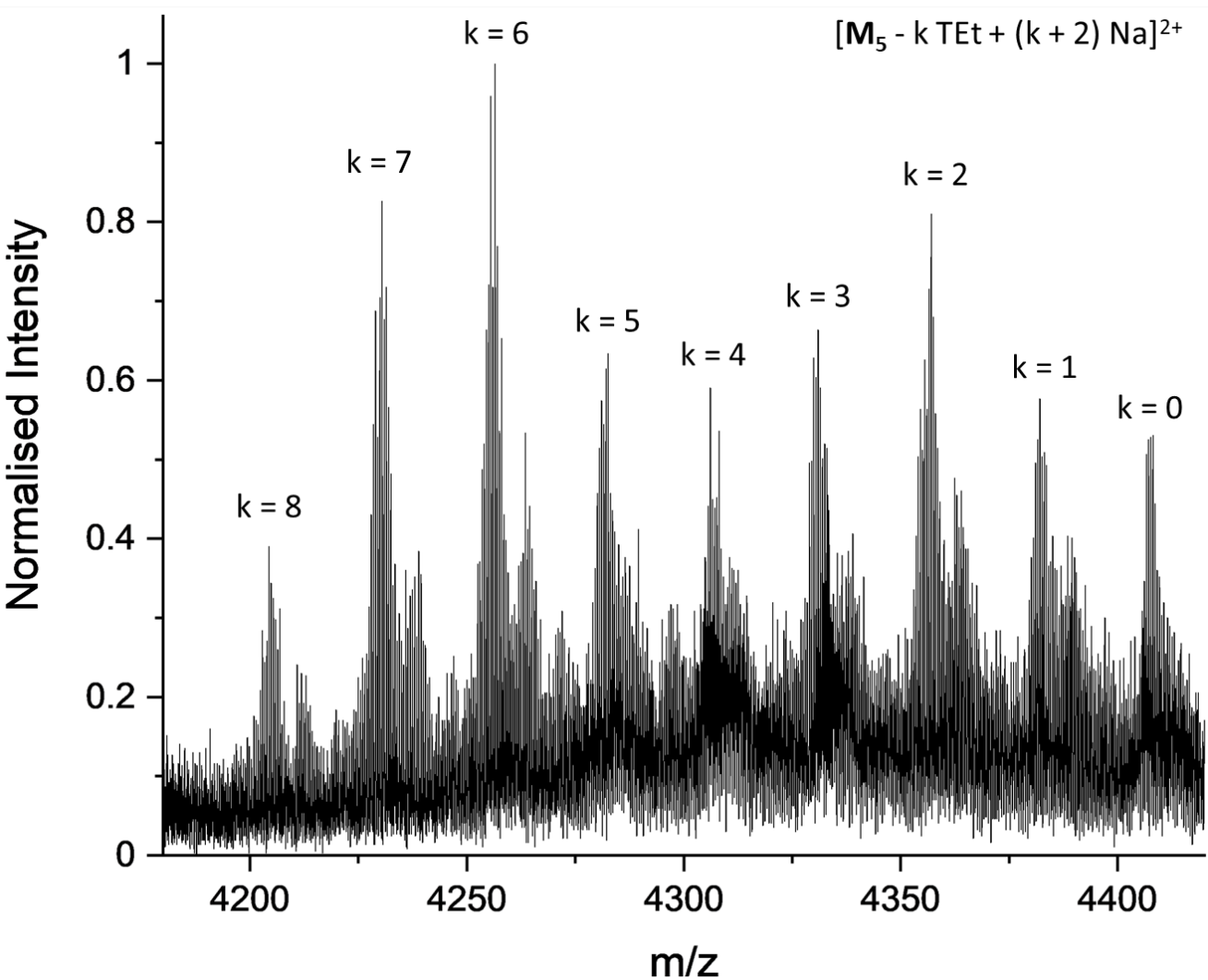


**Figure S1:** Zoom in the mass spectrum of **M** for m/z = 4200 – 4400. 200 µM **M** were transferred to the gas phase in a solution of 500 µM NaI and 4:1 toluene/methanol. **M** pentamers of the formula [**M_5_** – k TEt + (k + 2) Na]^2+^ (k = 0 – 8) were found in small amounts.


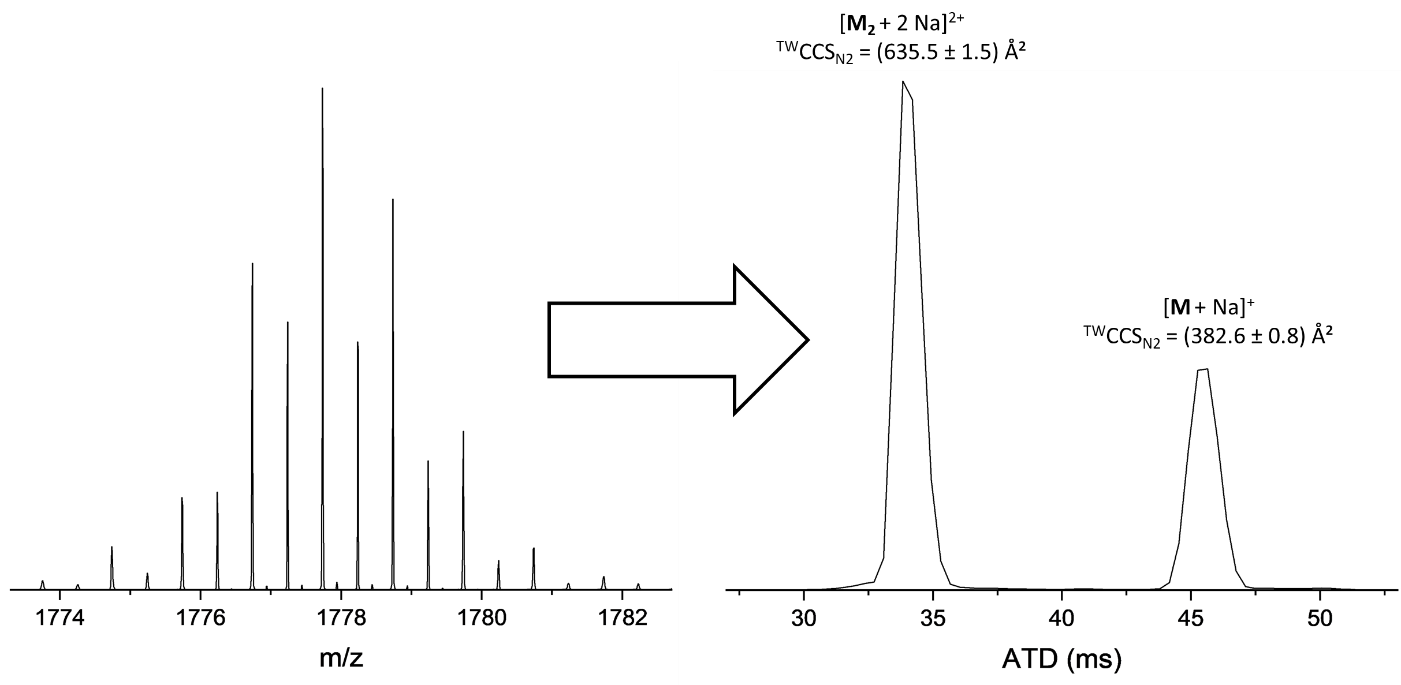


**Figure S2:** Mass spectrum and arrival time distribution (ATD) of [**M** + Na]^+^ and [**M_2_** + 2 Na]^2+^. Although the signals of both ions overlap in the mass spectrum, ion mobility allows their separation.

**Table S1:** ^TW^CCS_N2_ values of the ions [**M_n_** – k TEt + (k + z) Na]^z+^,
with n = 1 – 5, k = (-2) – 8 and z = 1 – 2.

| **n** | **z** | **k** | **m/z** | **^TW^CCS_N2_ in Å^2^ (Main Conformation)** | **^TW^CCS_N2_ in Å^2^ (Minor Conformation)** |
| --- | --- | --- | --- | --- | --- |
| 1 | 1 | 2 | 1675.3 | 368.0 ± 0.1 | - |
|  |  | 1 | 1726.4 | 374.6 ± 0.8 | - |
|  |  | 0 | 1777.5 | 382.6 ± 0.8 | - |
|  |  | -1 | 1828.6 | 398.1 ± 0.8 | - |
| 2 | 1 | 3 | 3378.7 | 559.8 ± 0.6 | - |
|  |  | 2 | 3429.8 | 574.0 ± 0.6 | - |
|  |  | 1 | 3480.9 | 575.5 ± 0.4 | - |
|  |  | 0 | 3532.0 | 614.4 ± 0.6 | - |
|  |  | -1 | 3583.1 | 626.6 ± 0.8 | - |
|  | 2 | 4 | 1675.3 | 566.8 ± 0.0 | 586.1 ± 0.8 |
|  |  | 3 | 1700.9 | 575.6 ± 1.6 | 609.1 ± 3.7 |
|  |  | 2 | 1726.4 | 591.2 ± 2.3 | 619.4 ± 0.4 |
|  |  | 1 | 1752.0 | 602.2 ± 1.7 | 633.8 ± 3.1 |
|  |  | 0 | 1777.5 | 635.5 ± 1.5 | - |
|  |  | -1 | 1803.1 | 643.9 ± 1.0 | - |
|  |  | -2 | 1828.6 | 654.8 ± 0.4 | - |
| 3 | 2 | 4 | 2552.6 | 750.0 ± 1.6 | 762.3 ± 5.0 |
|  |  | 3 | 2578.1 | 767.0 ± 1.2 | 774.7 ± 2.8 |
|  |  | 2 | 2603.7 | 777.8 ± 1.6 | 786.9 ± 1.0 |
|  |  | 1 | 2629.2 | 791.8 ± 1.2 | - |
|  |  | 0 | 2654.8 | 801.3 ± 0.8 | - |
|  |  | -1 | 2680.3 | 812.5 ± 0.5 | - |
|  |  | -2 | 2705.9 | 825.0 ± 0.6 | - |
| 4 | 2 | 6 | 3378.7 | 897.8 ± 0.7 | - |
|  |  | 4 | 3429.8 | 917.2 ± 0.3 | 894.5 ± 1.2 |
|  |  | 3 | 3455.3 | 935.4 ± 1.8 | - |
|  |  | 2 | 3480.9 | 939.5 ± 0.6 | - |
|  |  | 1 | 3506.4 | 952.9 ± 1.9 | - |
|  |  | 0 | 3532.0 | 967.3 ± 3.2 | - |
| 5 | 2 | 8 | 4204.9 | 1061.2 ± 5.1 | - |
|  |  | 7 | 4230.4 | 1069.2 ± 4.4 | - |
|  |  | 6 | 4256.0 | 1077.6 ± 4.5 | - |
|  |  | 5 | 4281.5 | 1087.8 ± 5.0 | - |
|  |  | 4 | 4307.1 | 1092.7 ± 3.9 | - |
|  |  | 3 | 4332.6 | 1099.6 ± 3.8 | - |
|  |  | 0 | 4409.8 | 1100.4 ± 5.6 | - |


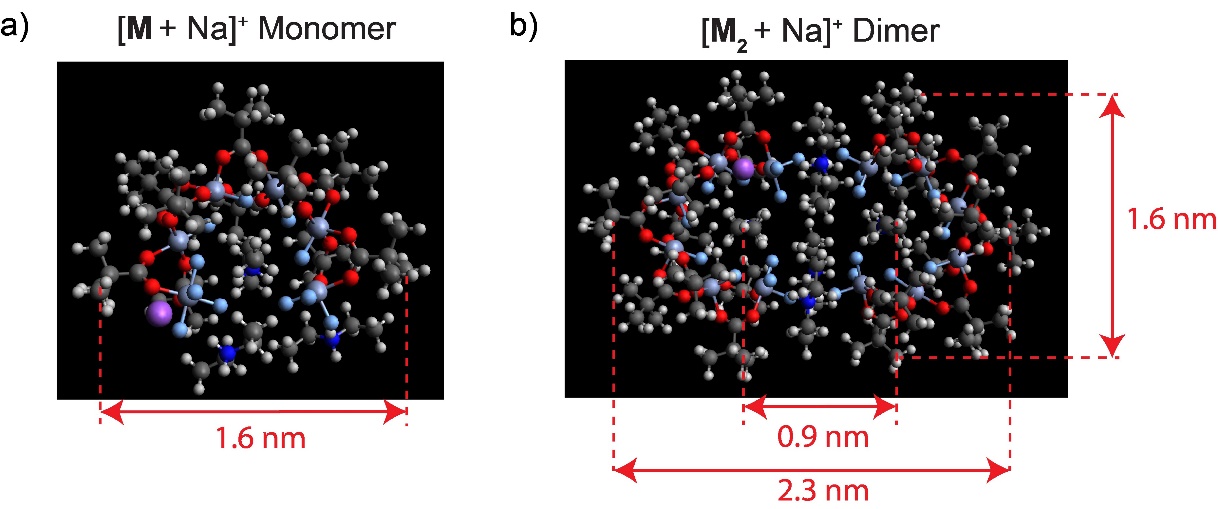


**Figure S3:** DFT optimised structures of a) [**M** + Na]^+^ and b) [**M_2_** + Na]^+^ including
distance measurements.


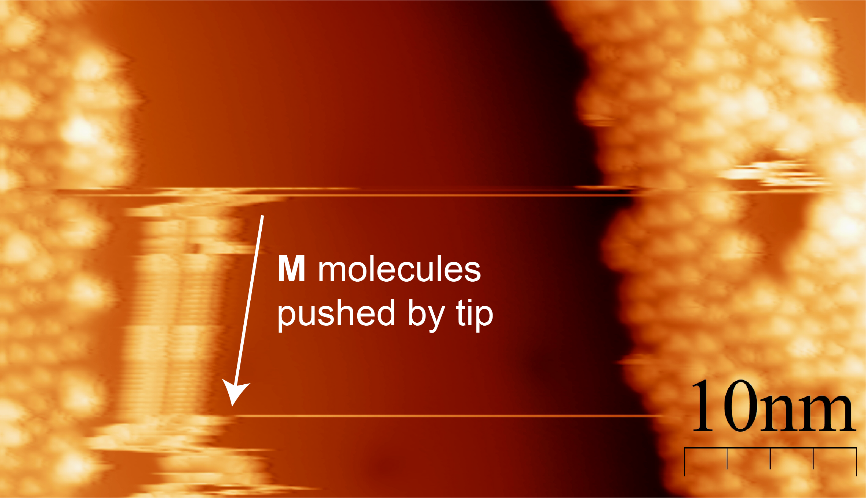


**Figure S4:** STM image of [**M_2_** + Na]^+^ on Ag(111) after STM tip manipulation. The surface was held at room temperature and imaged at 11 K. The constant-current STM imaging (Setpoint: I_t_ = 5 pA, V_b_ = 300 mV) shows isolated **M** molecules (molecules that are not in the sub-monolayer) being pushed by the STM tip during the raster scanning, demonstrating that **M** is easily perturbed and can move on the Ag(111) surface at 11 K.

**Table S2:** Measurement of features **A** and **B** as observed in Figure 3. The dimensions of **A** were estimated by measuring and averaging the distance between two adjacent features. The dimensions of **B** were estimated by measuring and averaging the distances between maxima within a given feature (exemplified by yellow dashed lines in Figure 3c and Figure 3g). Uncertainties listed correspond to standard deviations of the measured distances.

|  | [**M_2_** + Na]^+^ on Ag(111) | [**M_3_** + 2 Na]^2+^ on Ag(111) |
| --- | --- | --- |
| **A** | Along **r_1_** : 1.72 ± 0.06 nm (*N = 10*)  Along **r_2_** : 1.71 ± 0.05 nm (*N = 11*) | 1.72 ± 0.17 nm (*N* = 12) |
| **B** | 0.79 ± 0.06 nm (*N* = 4) | 0.75 ± 0.09 nm (*N* = 10) |


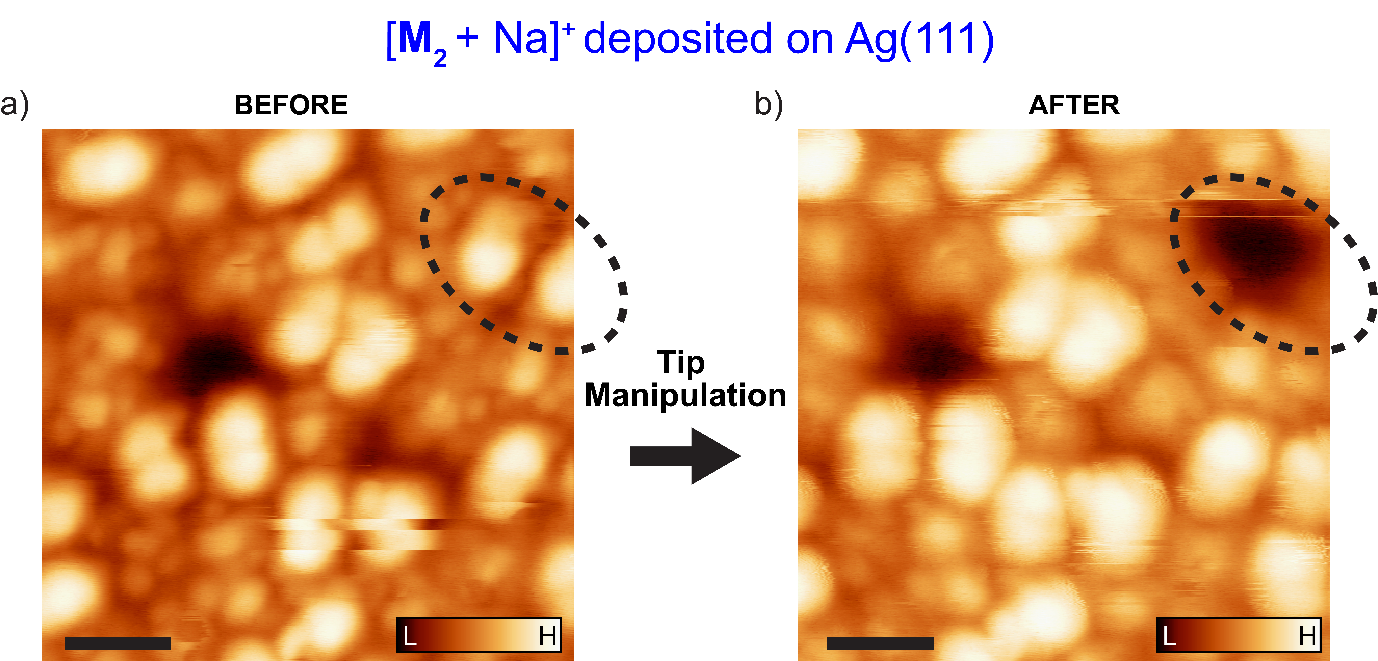


**Figure S5:** STM tip manipulation and removal of **M_4_**. a) Constant-current STM imaging of a **M** self-assembled island on Ag(111) (Setpoint: I_t_ = 0.1 pA, V_b_ = 300 mV) upon deposition of [**M_2_** + Na]^+^. b) Constant-current STM imaging of the same area in a) after the STM tip was used to remove the dimer-dimer pair indicated (black dashed line) in a). The resulting area where the dimer of dimer used to reside shows no presence of **M** monomers, indicating that the **M_4_** dimers of dimers were part of the first layer of the **M** self-assembled islands alongside the **M** monomers. Interestingly, the STM tip was used to remove one **M_2_** dimer but the entire **M_4_** dimer of dimer was removed, suggesting a strong dimer-dimer interaction. Scale bars: 2 nm.


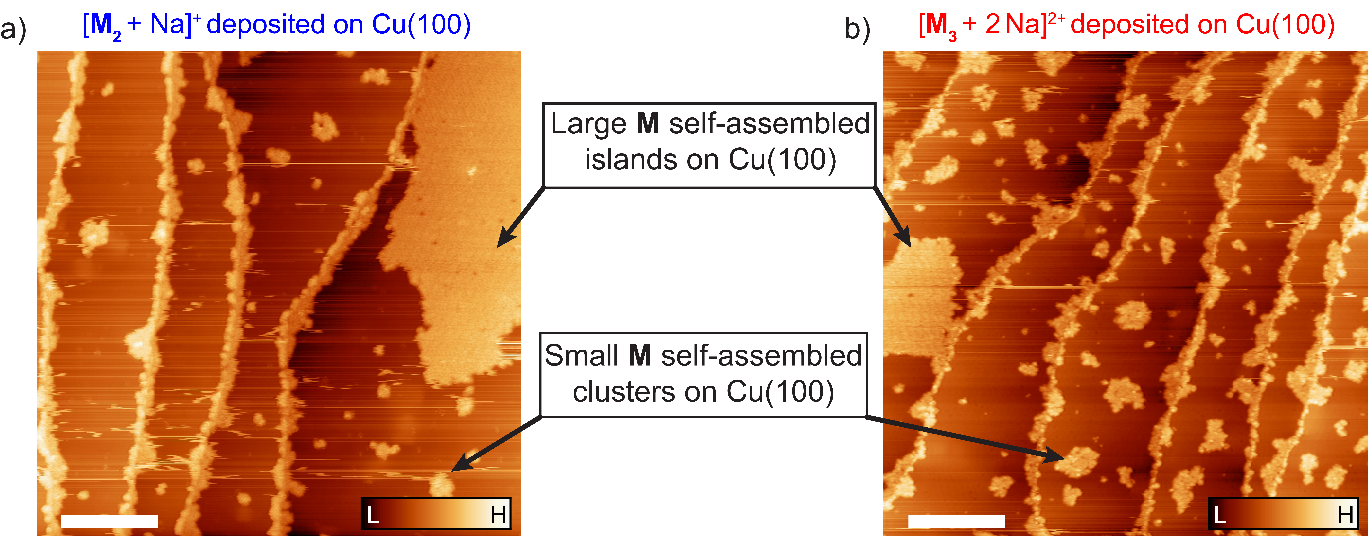


**Figure S6:** STM images for the deposition on Cu(100). a) STM image of [M2 + Na]+ on Cu(100). Constant-current STM imaging (Setpoint: It = 0.1 pA, Vb = 300 mV) of a Cu(100) surface after deposition of [**M_2_** + Na]^+^ (*m/z* = 3532) onto the surface held at room temperature. b) STM image of [**M_3_** + 2 Na]^2+^ on Cu(100). Constant-current STM imaging (Setpoint: I_t_ = 0.1 pA, V_b_ = 300 mV) of a Cu(100) surface after deposition of [**M_3_** + 2 Na]^2+^ (*m/z* = 2655) onto the surface held at room temperature. In both cases, we observed the formation of large **M** self-assembled islands on Cu(100) in addition to many smaller **M** self-assembled clusters on Cu(100). STM images were recorded at 11 K. Scale bars: 40 nm.


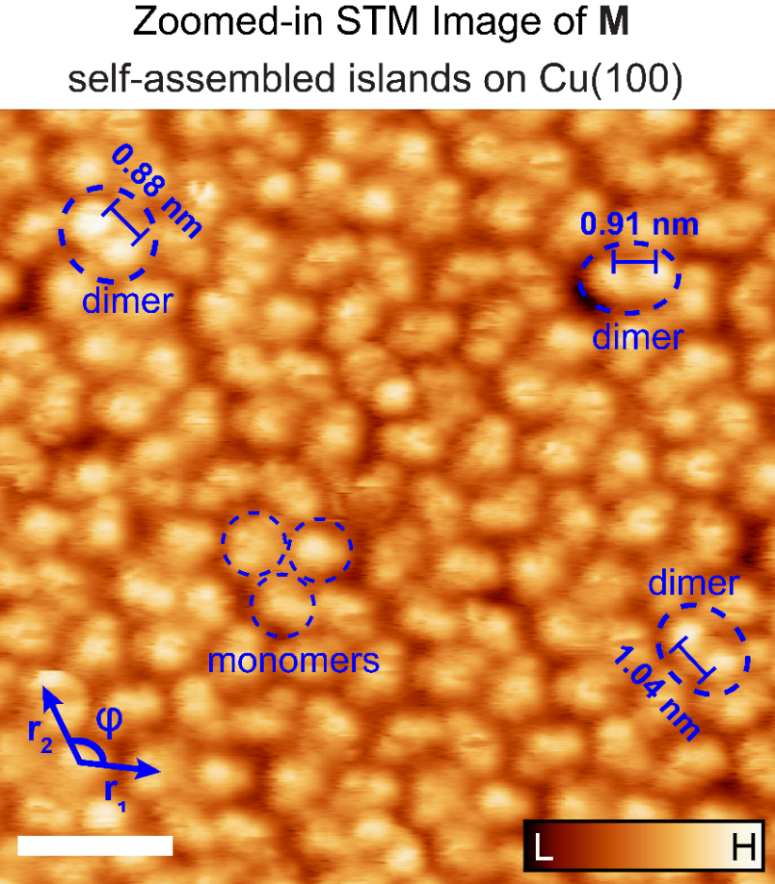


**Figure S7:** STM image including structural annotations for the deposition of [**M_3_** + 2 Na]^2+^ on Cu(100). Constant-current STM imaging of a zoomed-in **M** self-assembled island on Cu(100) (Setpoint: I_t_ = 0.1 pA, V_b_ = 300 mV) upon deposition of [**M_3_** + 2 Na]^2+^. Unlike on Ag(111), the resulting 2D self-assembled islands on Cu(100) are only partially ordered with approximate lattice vectors of lengths $|\mathbf{r}_{\mathbf{1}}\boldsymbol{|}$ = 1.86 ± 0.15 nm and $|\mathbf{r}_{\mathbf{2}}\boldsymbol{|}$ = 1.73 ± 0.11 nm separated by an angle of $\varphi$= 122 ± 2°. The partial ordering of the islands is reflected in the random orientations of the individual features observed in the islands on Cu(100) which can be explained by the high diffusion barriers that the molecules encounter on Cu(100). We do, however, observe that the islands are composed of **M** monomers and **M_2_** dimers, but not **M_4_** dimers of dimers as observed on Ag(111). We further note that the assignment of **M** monomers and **M_2_** dimers to the features observed on Cu(100) is not as well-defined as on Ag(111), and that that the **M_2_** dimers do not have a larger apparent height as observed on Ag(111). Scale bars: 4 nm.


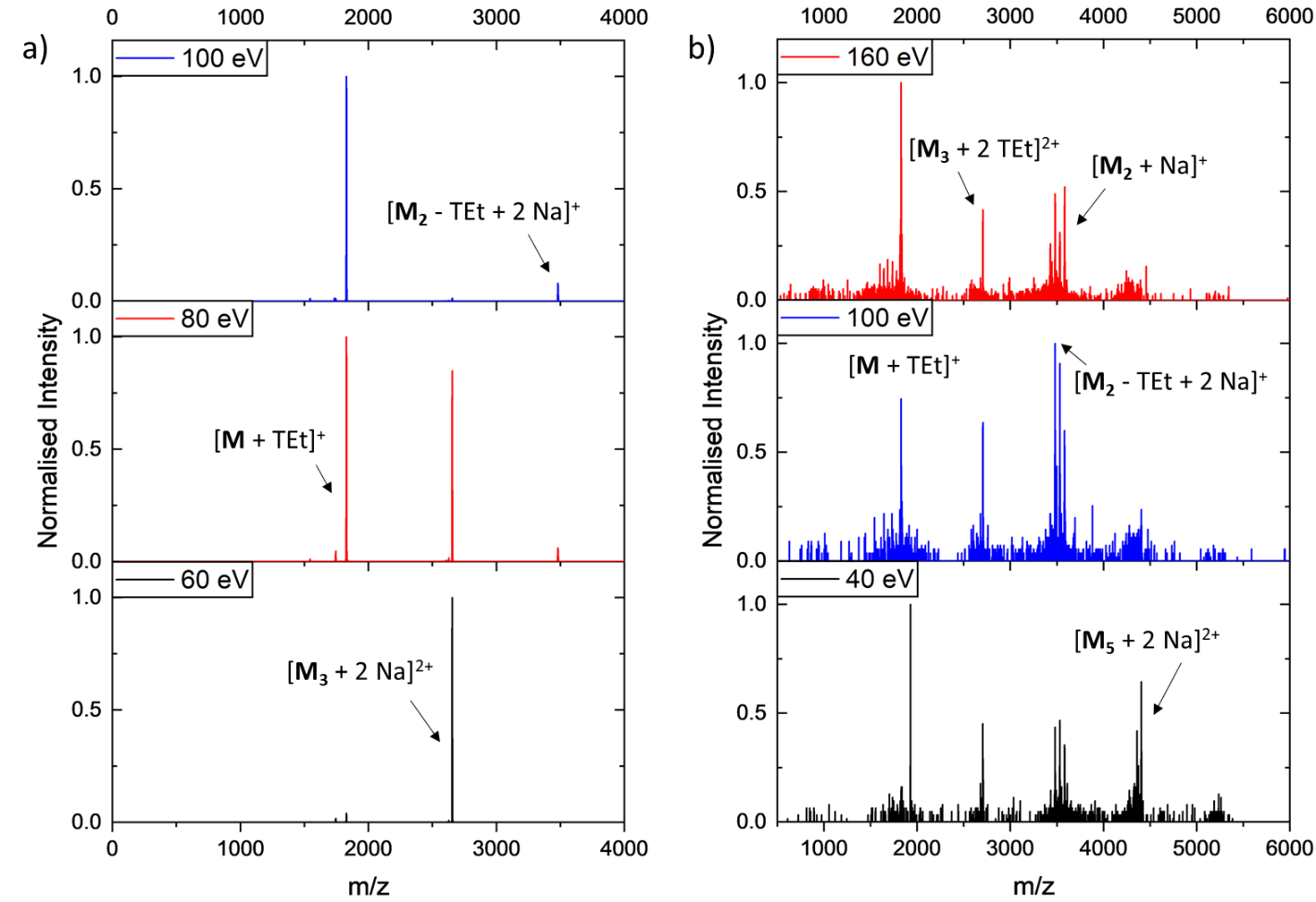


**Figure S8:** MS^2^ spectra of a) [**M_3_** + 2 Na]^2+^ at E_lab_ = 60 – 100 eV and b) [**M_5_** + 2 Na]^2+^ at E_lab_ = 40 – 160 eV. Ions were isolated at m/z = 2655 and m/z = 4406, respectively. Both precursor ions show an overall tendency to dissociate to TEt^+^-rich monomers/trimers and Na^+^-rich dimers. For a), we attribute the significantly higher intensity of [**M** + TEt]^+^, compared to
[**M_2_** – TEt + Na]^+^, to a subsequent fragmentation of the dimeric ion to [**M** + TEt]^+^ and the neutral unit [**M** – 2 TEt + 2 Na]. The alternative explanation, the fragmentation of the precursor ion to three monomers, can be excluded as the main fragment ion [**M** + TEt]^+^ does not contain any Na^+^ that is present in the precursor ion.


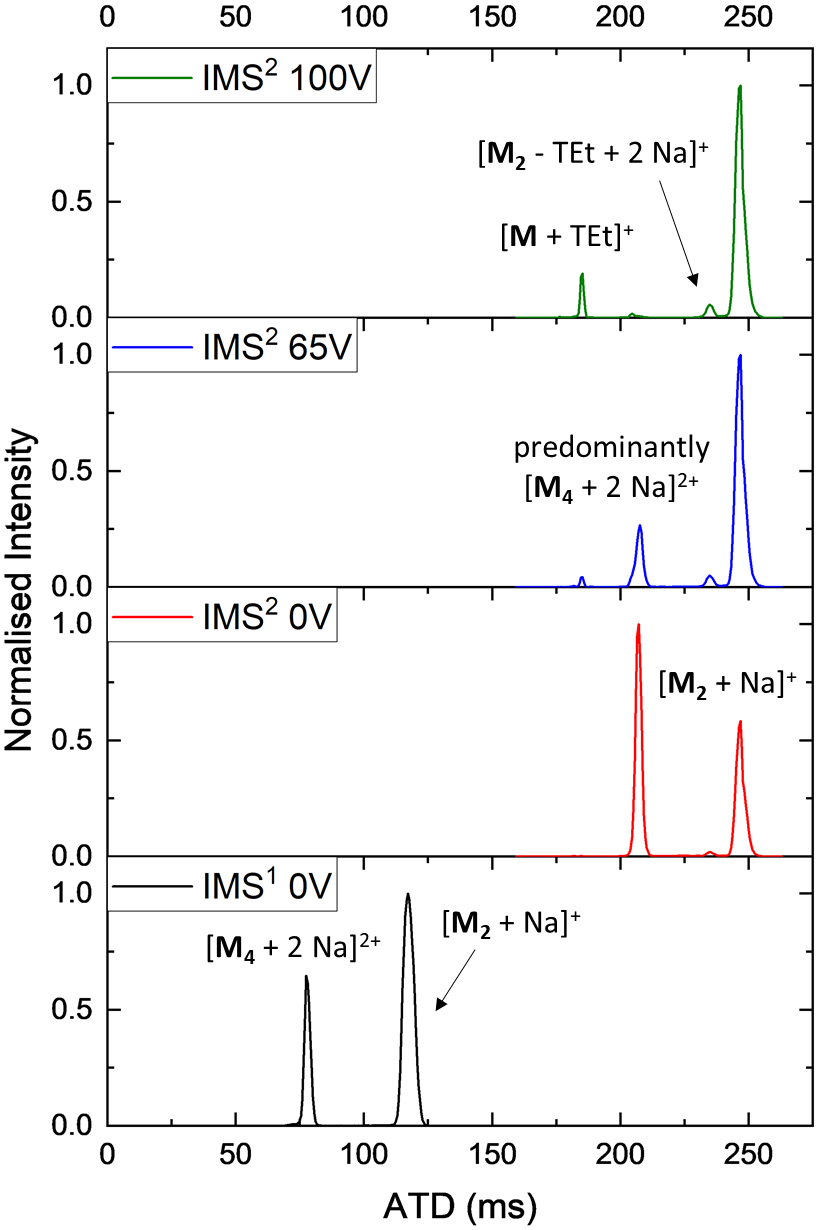


**Figure S9:** ATDs of the ions [**M_2_** + Na]^+^ and [**M_4_** + 2 Na]^2+^ at various collision energies
(m/z = 3532). The dimer appears as the prevalent ion (IMS^1^ 0V), however after mobility-selection of [**M_4_** + 2 Na]^2+^, a significant amount of the tetramer dissociates to the dimer without further collisional activation (IMS^2^ 0V). The homolytic dissociation to the product
[**M_2_** + Na]^+^ appears as the main fragmentation channel as well after collisional activation (IMS^2^ 65V and IMS^2^ 100V).


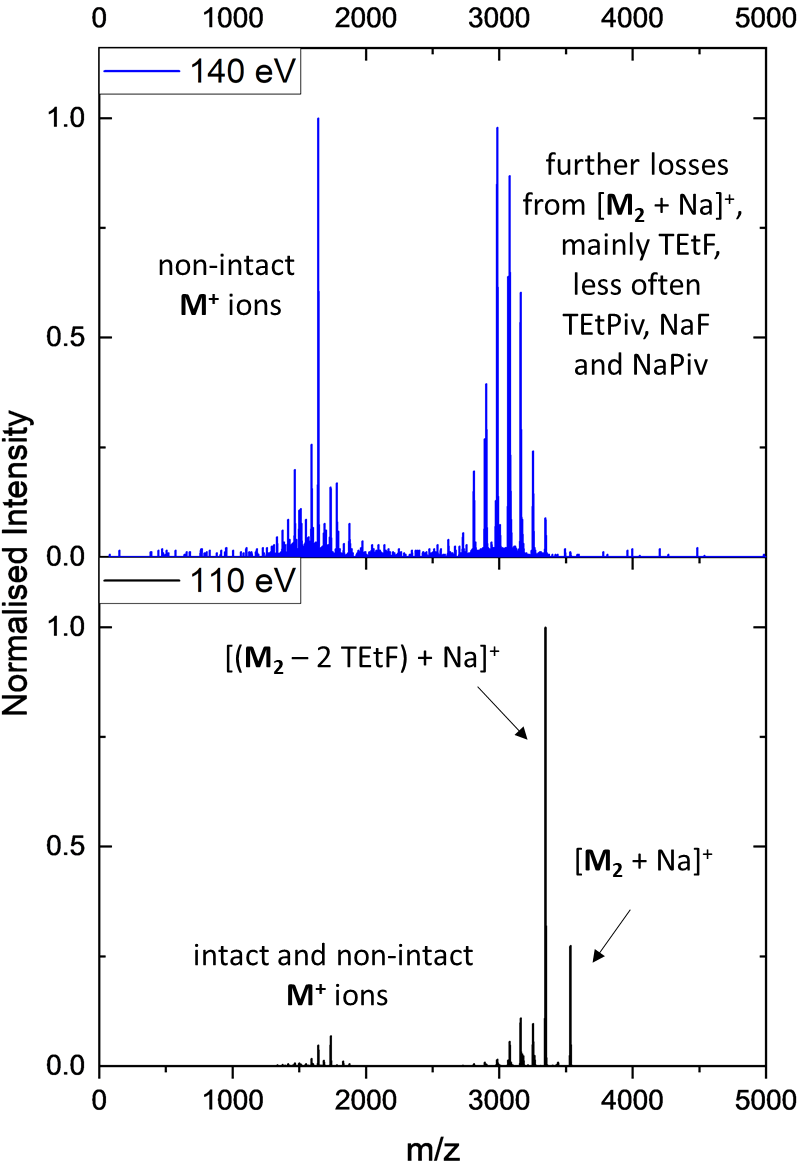


**Figure S10:** MS^2^ spectra of [**M_2_** + Na]^+^ at E_lab_ = 110 eV (bottom) and E_lab_ = 140 eV (top). Ions were isolated at m/z = 3532. Although there is an overlap with the species [**M_4_** + 2 Na]^2+^ in the mass spectrum, no mobility selection was applied because the collision-induced dissociation in the IMS^2^ experiment is not sufficient to fragment [**M_2_** + Na]^+^. However, the amount of
[**M_4_** + 2 Na]^2+^ in the precursor population was negligible in this case (Supplementary Dataset). The main dissociation pathway involves the loss of TEtF units, before the non-intact **M_2_^+^** cluster dissociate to non-intact **M^+^** ions at higher energies (top).


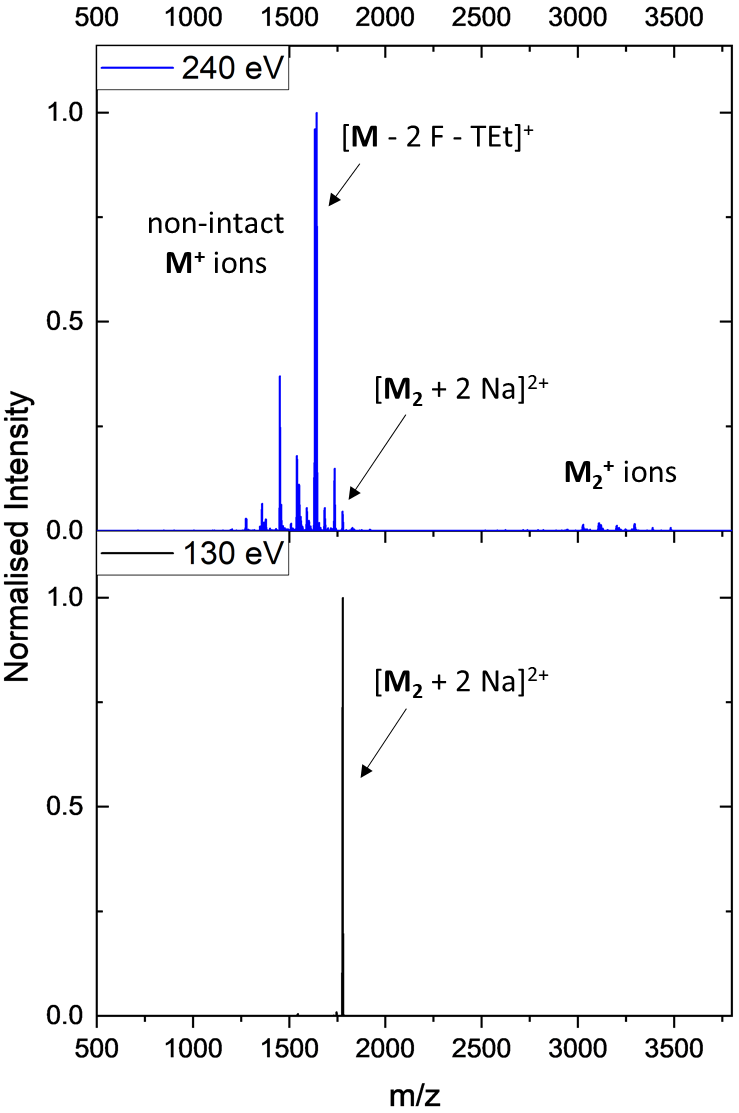


**Figure S11:** MS^2^ spectra of [**M_2_** + 2 Na]^2+^ at E_lab_ = 130 eV (bottom) and E_lab_ = 240 eV (top). Ions were isolated at m/z = 1777 and mobility-selected (IMS^2^). [**M_2_** + 2 Na]^2+^ mainly dissociates to non-intact **M^+^** ions that predominantly lost TEtF and NaF units. Small amounts of the cluster type **M_2_^+^** were also found.


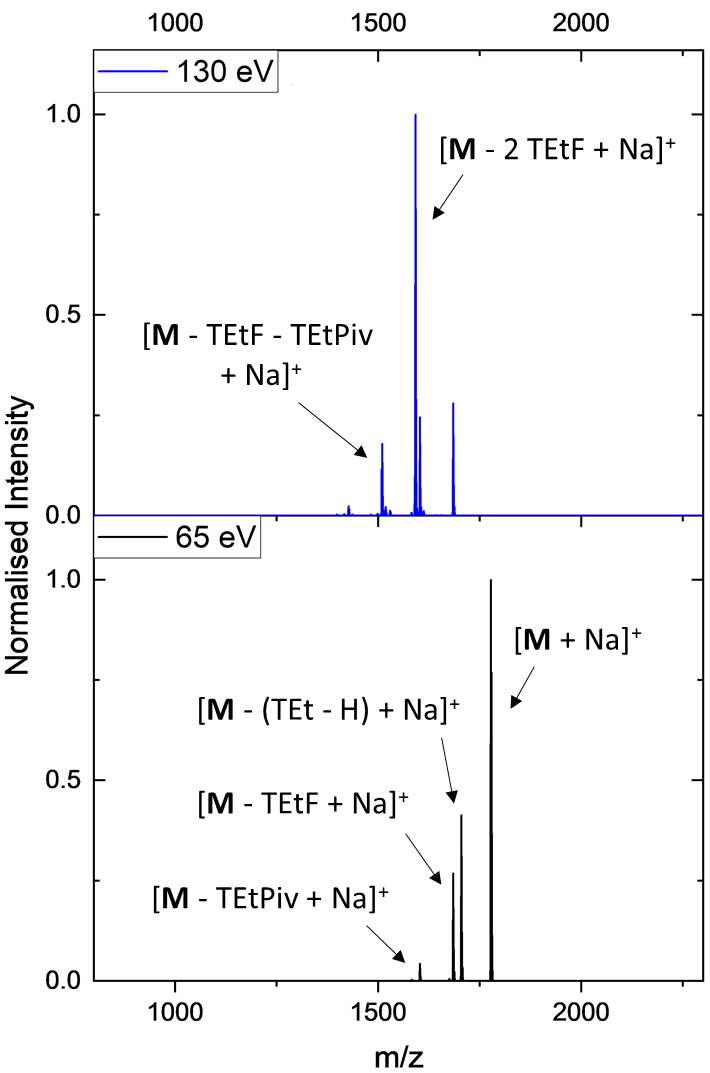


**Figure S12:** MS^2^ spectra of [**M** + Na]^+^ at E_lab_ = 65 eV (bottom) and E_lab_ = 130 eV (top). Ions were isolated at m/z = 1778 and mobility-selected (IMS^2^). [**M** + Na]^+^ fragments via losses of TEtF, TEtPiv and the deprotonated thread [TEt – H] = NH(CH_2_CH_3_)_2_.


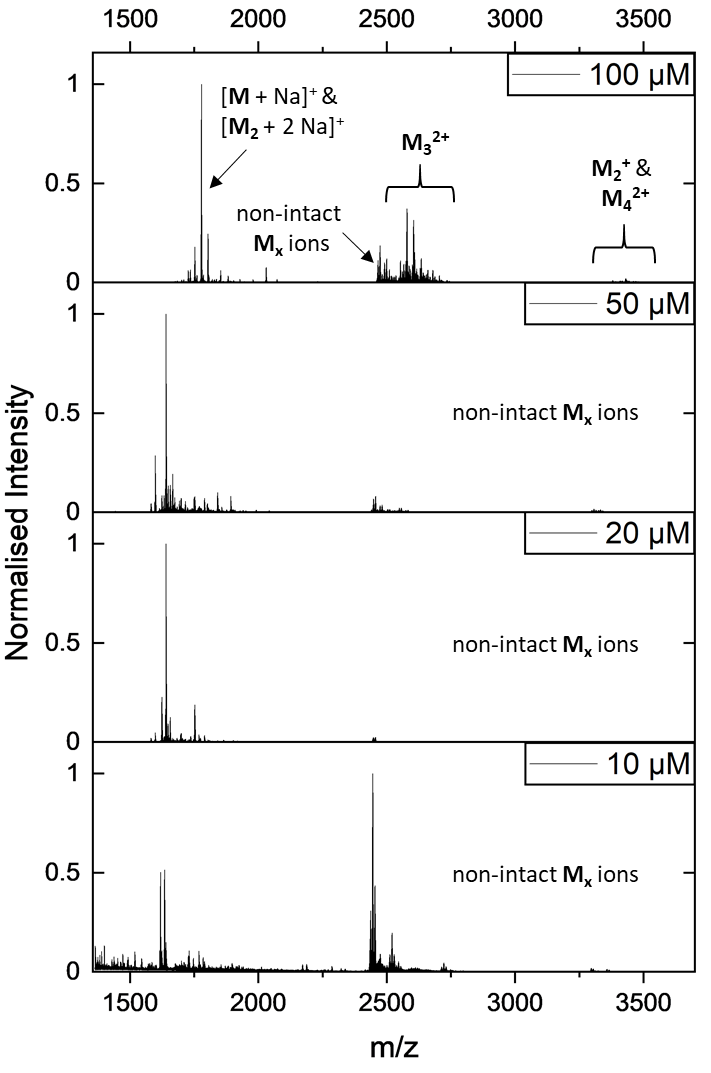


**Figure S13:** Mass spectra of **M** at concentrations = 10 – 100 µM. All samples were sprayed from 500 µM NaI and 4:1 toluene/methanol. Intact **M** ions were observed at 100 µM, whereas almost no such ions were found at lower concentrations. Different ratios of intact/non-intact **M** ions and charge state distributions were obtained from day to day and under varying instrument conditions. However, lower **M** concentrations generally yielded less intact **M** ions, likely due to an entropic threshold in the self-assembly of **M** itself.

**References**

[1] F. K. Larsen, J. Overgaard, S. Parsons, E. Rentschler, A. A. Smith, G. A. Timco, R. E. P. Winpenny, *Angew. Chem. Int. Ed.* **2003**, *42*, 5978–5981.

[2] K. Giles, J. Ujma, J. Wildgoose, S. Pringle, K. Richardson, D. Langridge, M. Green, *Anal. Chem.* **2019**, *91*, 8564–8573.

[3] A. A. Shvartsburg, R. D. Smith, *Anal. Chem.* **2008**, *80*, 9689–9699.

[4] D. Morsa, V. Gabelica, E. De Pauw, *Anal. Chem.* **2011**, *83*, 5775–5782.

[5] S. I. Merenbloom, T. G. Flick, E. R. Williams, *J. Am. Soc. Mass Spectrom.* **2012**, *23*, 553–562.

[6] B. T. Ruotolo, J. L. P. Benesch, A. M. Sandercock, S. J. Hyung, C. V. Robinson, *Nat. Protoc.* **2008**, *3*, 1139–1152.

[7] S. M. Stow, T. J. Causon, X. Zheng, R. T. Kurulugama, T. Mairinger, J. C. May, E. E. Rennie, E. S. Baker, R. D. Smith, J. A. McLean, S. Hann, J. C. Fjeldsted, *Anal. Chem.* **2017**, *89*, 9048–9055.

[8] M. J. Frisch, G. W. Trucks, H. B. Schlegel, G. E. ; Scuseria, M. A. ; Robb, J. R. ; Cheeseman, G. ; Scalmani, V. ; Barone, G. A. ; Petersson, H. ; Nakatsuji, X. ; Li, M. ; Caricato, A. V. ; Marenich, J. ; Bloino, B. G. ; Janesko, R. ; Gomperts, B. ; Mennucci, H. P. ; Hratchian, J. V. ; Ortiz, A. F. ; Izmaylov, J. L. ; Sonnenberg, D. ; Williams-Young, F. ; Ding, F. ; Lipparini, F. ; Egidi, J. ; Goings, B. ; Peng, A. ; Petrone, T. ; Henderson, D. ; Ranasinghe, V. G. ; Zakrzewski, J. ; Gao, N. ; Rega, G. ; Zheng, W. ; Liang, M. ; Hada, M. ; Ehara, K. ; Toyota, R. ; Fukuda, J. ; Hasegawa, M. ; Ishida, T. ; Nakajima, Y. ; Honda, O. ; Kitao, H. ; Nakai, T. ; Vreven, K. ; Throssell, J. A. Jr. ; Montgomery, J. E. ; Peralta, F. ; Ogliaro, M. J. ; Bearpark, J. J. ; Heyd, E. N. ; Brothers, K. N. ; Kudin, V. N. ; Staroverov, T. A. ; Keith, R. ; Kobayashi, J. ; Normand, K. ; Raghavachari, A. P. ; Rendell, J. C. ; Burant, S. S. ; Iyengar, J. ; Tomasi, M. ; Cossi, J. M. ; Millam, M. ; Klene, C. ; Adamo, R. ; Cammi, J. W. ; Ochterski, R. L. ; Martin, K. ; Morokuma, O. ; Farkas, J. B. ; Foresman, D. J. Fox, **2016**.

[9] S. Grimme, J. Antony, S. Ehrlich, H. Krieg, *J. Chem. Phys.* **2010**, *132*, 154104.

[10] P. J. Hay, W. R. Wadt, *J. Chem. Phys.* **1985**, *82*, 299–310.

[11] V. Shrivastav, M. Nahin, C. J. Hogan, C. Larriba-Andaluz, *J. Am. Soc. Mass Spectrom.* **2017**, *28*, 1540–1551.

[12] S. Rauschenbach, M. Ternes, L. Harnau, K. Kern, *Annu. Rev. Anal. Chem.* **2016**, *9*, 473–498.

[13] V. Grill, J. Shen, C. Evans, R. G. Cooks, *Rev. Sci. Instrum.* **2001**, *72*, 3149–3179.

[14] K. Anggara, Y. Zhu, M. Delbianco, S. Rauschenbach, S. Abb, P. H. Seeberger, K. Kern, *J. Am. Chem. Soc.* **2020**, *142*, 21420–21427.
